# Supplementary material for: Reversible On-Off Photoswitching of DNA Replication Using a Dumbbell Oligodeoxynucleotide
Source: Molecules. 2022 Dec 16;27(24):8992. doi: 10.3390/molecules27248992 (PMC9785685; doi:10.3390/molecules27248992)
Supplement: Supplementary file 1 [file molecules-27-08992-s001.zip › molecules-2050250-supplementary.pdf]

Supporting Information

Reversible on-off Photoswitching of DNA Replication Using a Dumbbell Oligodeoxynucleotide

Yu Wang [1,†], Heming Ji [1,2,†], Jian Ma [1,†], Hang Luo [1], Yujian He [1], Xinjing Tang [3,*] and Li Wu [1,2,*]

[1] School of Chemical Sciences, University of Chinese Academy of Sciences, Beijing 100049, China
[2] Zhejiang Institute of Mechanical and Electrical Technician, Yiwu 322000, China
[3] State Key Laboratory of Natural and Biomimetic Drugs, School of Pharmaceutical Sciences, Peking University, Beijing 100191, China
* Correspondence: xinjingt@bjmu.edu.cn (X.T.); wuli@ucas.ac.cn (L.W.)
† These authors contributed equally to this work.

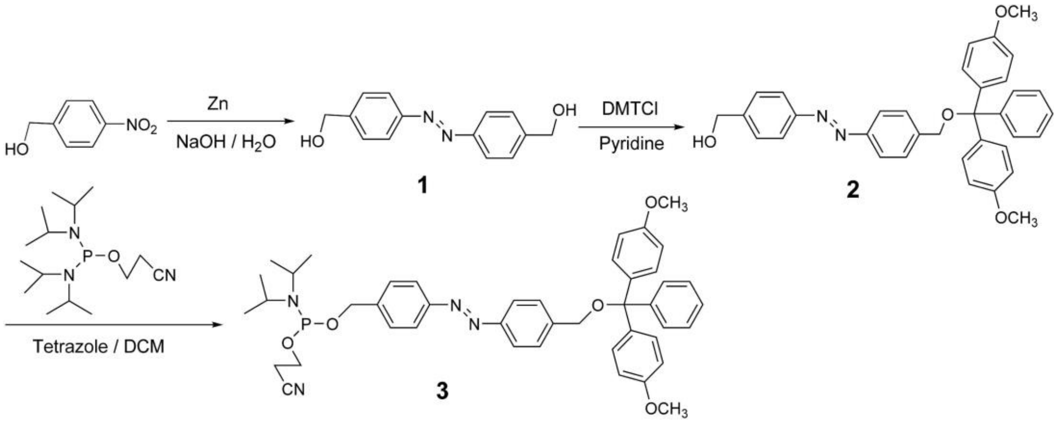

Figure S1. Synthetic procedure of azobenzene phosphoramidite.

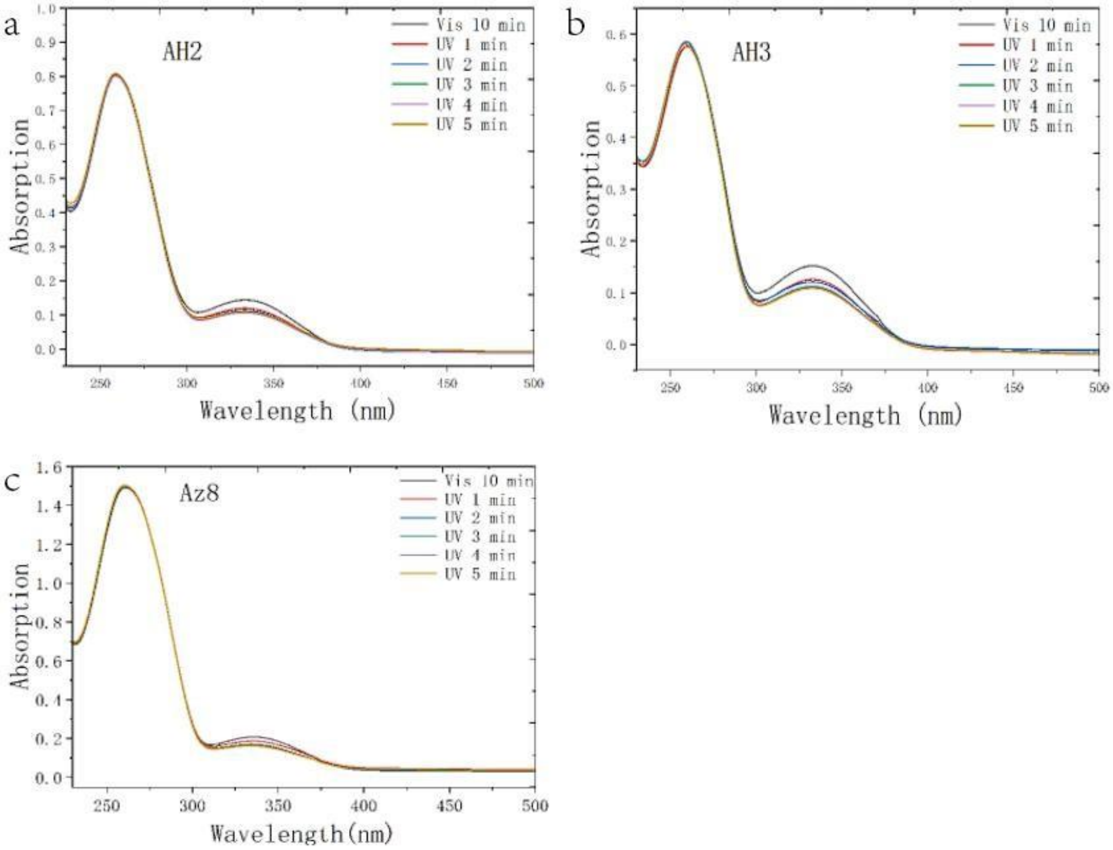

Figure S2. Photoisomerization of asODNs and azODNs. After a wavelength illumination time of 365 nm for 1 min, 2 min, 3 min, 4 min and 5 min, the characteristic absorption peak at asODNs and azODNs 35nm decreased. **a** AAAAGazoCTTTT; **b** AATAGazoCTATT; **c** 5’-GTTGGazoCCAACGTTTCGGACCGTATTazoAATAC-3'.

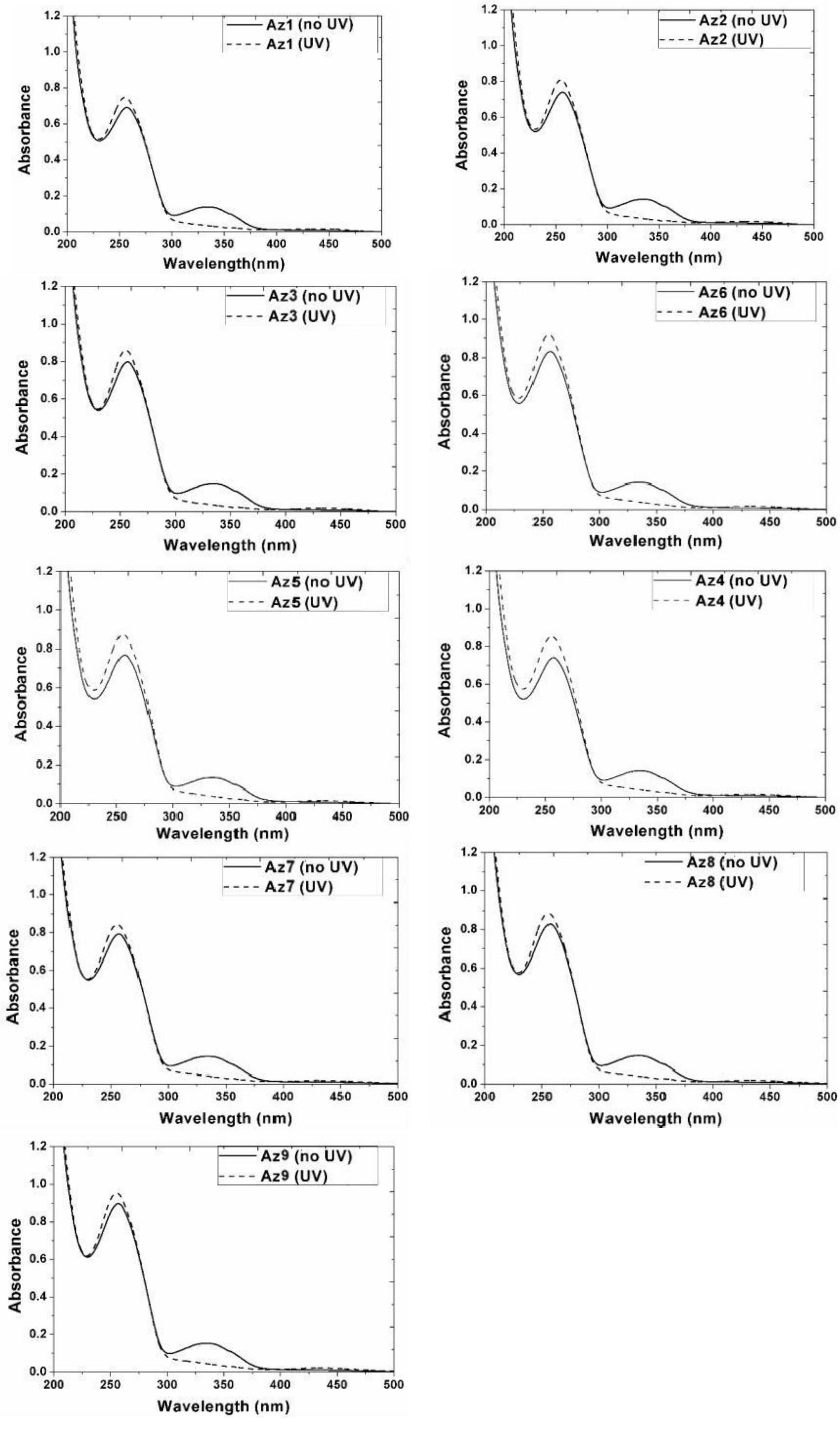
Az1 (no UV)
Az1 (UV)
Az2 (no UV)
Az2 (UV)
Az3 (no UV)
Az3 (UV)
Az6 (no UV)
Az6 (UV)
Az5 (no UV)
Az5 (UV)
Az4 (no UV)
Az4 (UV)
Az7 (no UV)
Az7 (UV)
Az8 (no UV)
Az8 (UV)
Az9 (no UV)
Az9 (UV)
Absorbance
Wavelength(nm)
Wavelength (nm)
0.0
0.2
0.4
0.6
0.8
1.0
1.2
200
250
300
350
400
450
500

Figure S3. UV/vis absorbance spectra of the respective isomeric forms of all azobenzene linked dumbbell ODNs before and after light illumination for 30 min. The samples (2 μM) were irradiated with UV (365 nm, trans-to-cis) in 1× PBS until absorbance changes were no longer observable.

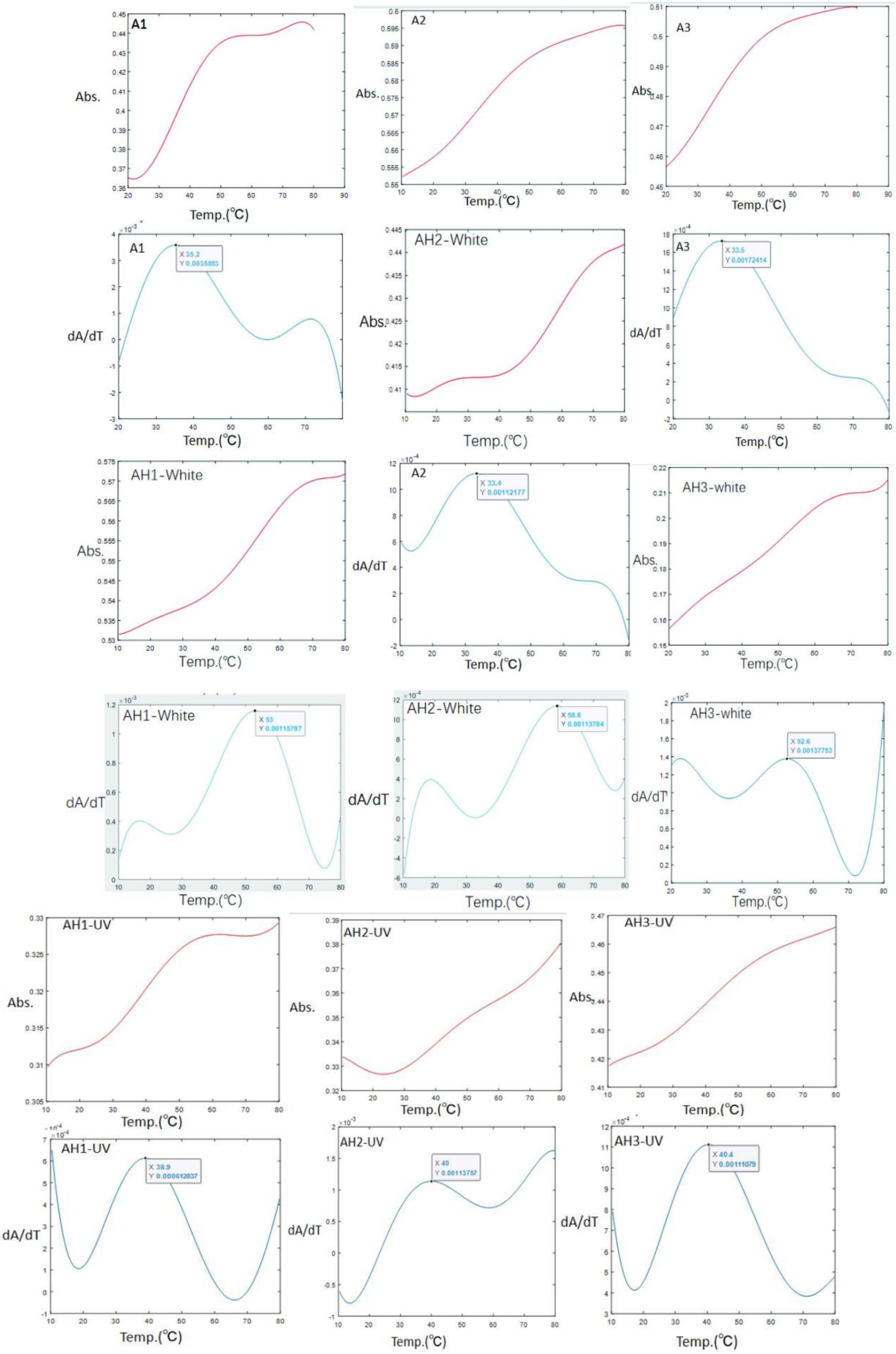

Figure S4 Measure the hairpin azODNs dissolution curve with a variable temperature UV spectrophotome ter. AH1, AH2 and AH3 solutions were obtained by detecting the absorption value of 260 nm after 30 m inutes of UV or white light illumination, respectively. The matrix software uses the derivative of the disc rete function difference to the a-discrete test value.

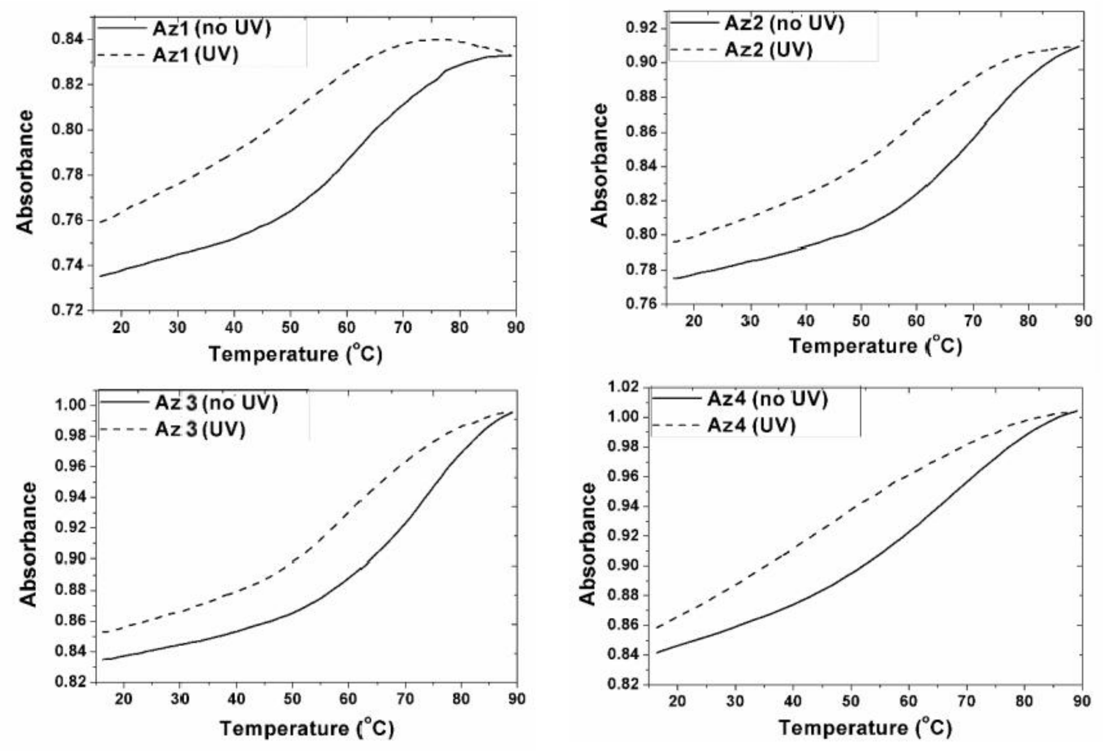

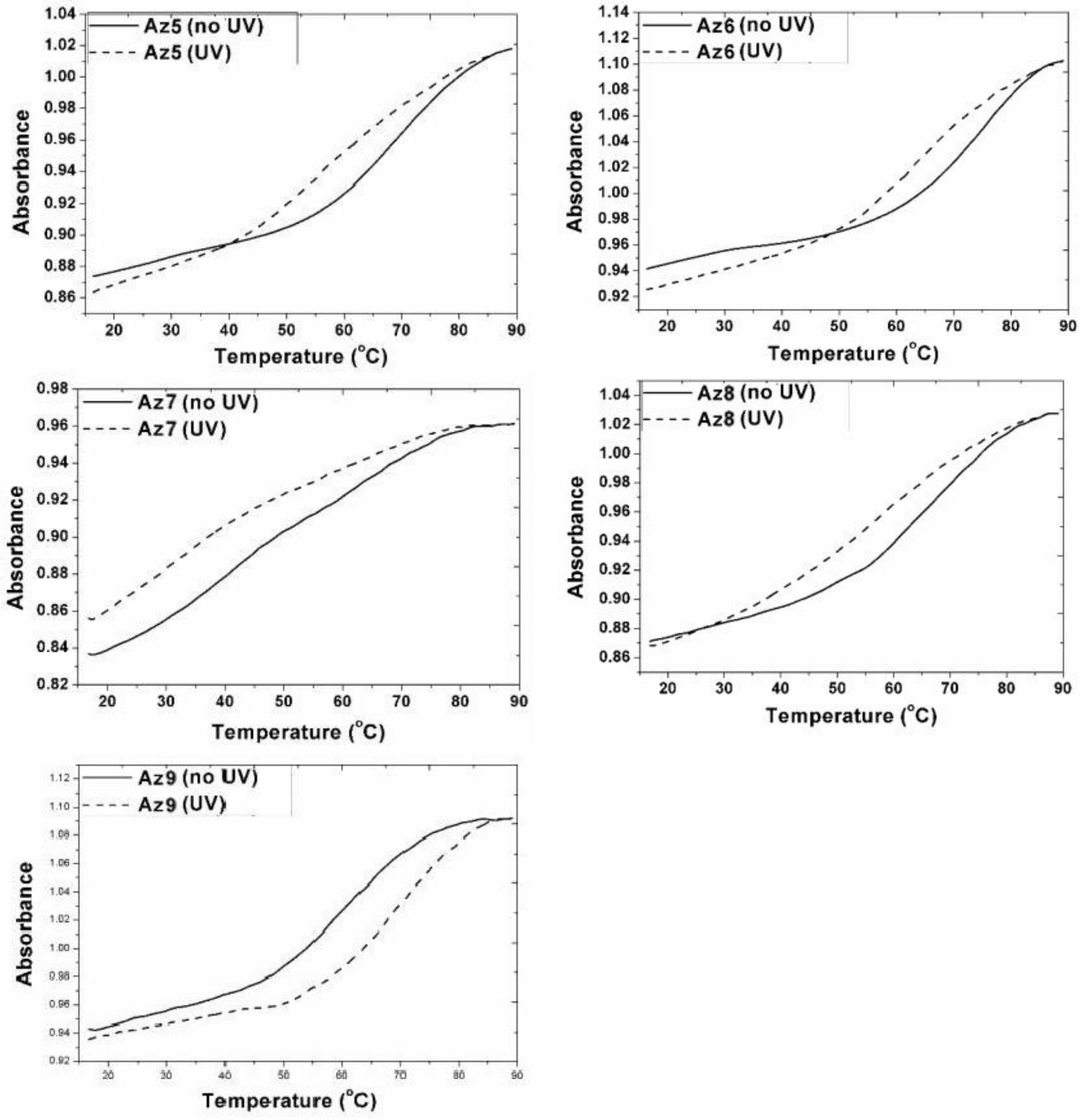

Figure S5 Typical melting curves of all azobenzene linked dumbbell asODNs themselves, and the black and dashed lines present the melting curves before and after irradiation with UV light (365 nm), respectively. UV melting experiments were performed in 1× PBS and ODN concentrations were adjusted to 2 μM.

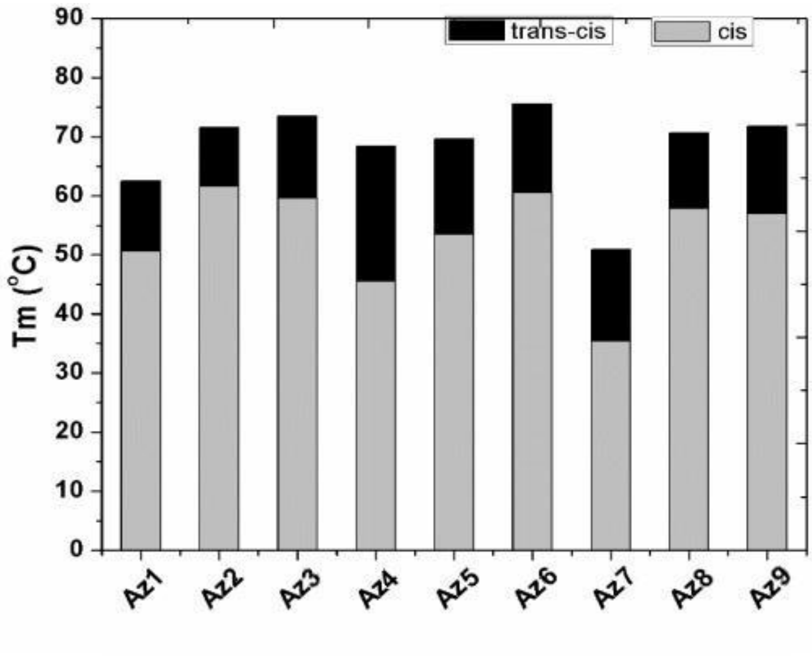

Figure S6. T_m comparison of azobenzene linked dumbbell asODNs before and after UV irradiation. Light grayrepresents T_m of cis-azobenzene linked ODNs, and black represents T_m of trans-azobenzene linked ODNs deducting that of the corresponding cis conformation.

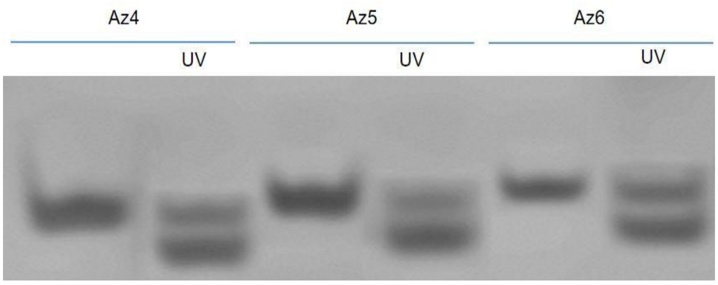

Figure S7 Comparison of natural polyacrylamide gel electrophoresis strips with light azobenzene linked to dumbbell nucleic acid (365 nm). The samples from left to right were corresponding to trans-Az4, cis-Az4, trans-Az5 cis-Az5, trans-Az6, cis-Az6.

^{1}H NMR Spectra of Compound 1

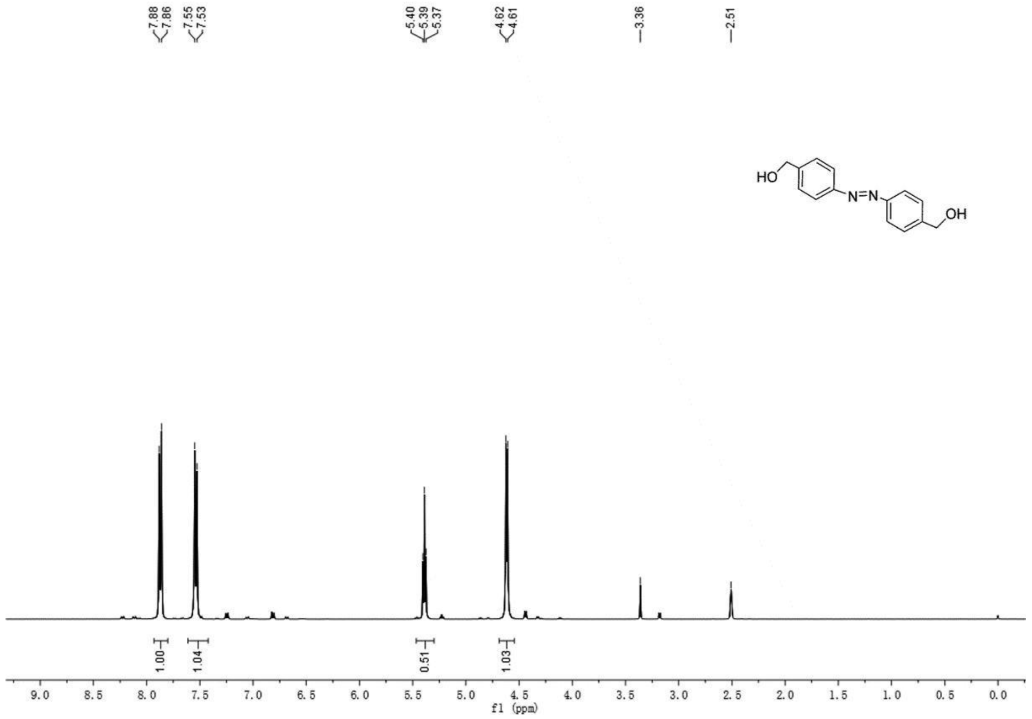

[13]c NMR Spectra of Compound 1

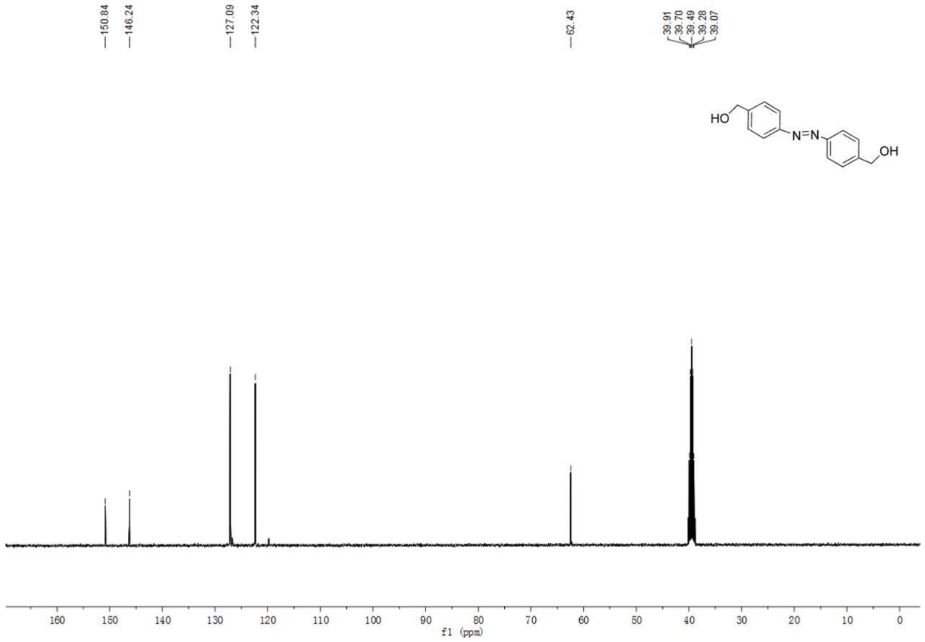

^{1}H NMR Spectra of Compound 2

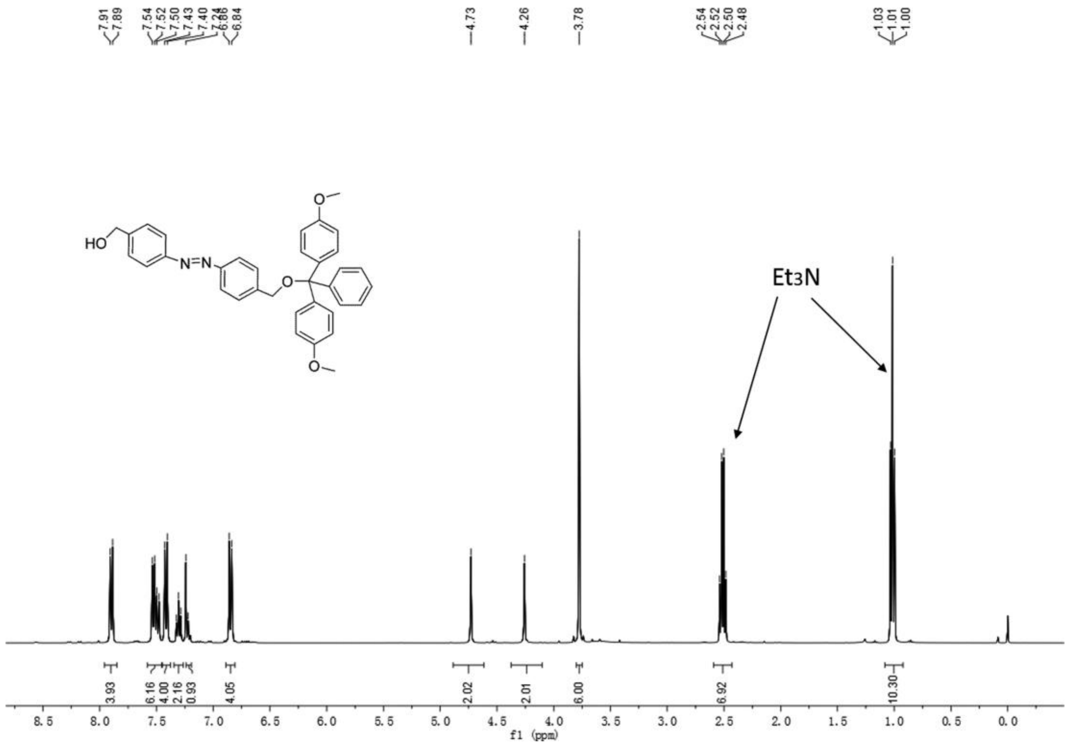

^{13}C NMR Spectra of Compound 1

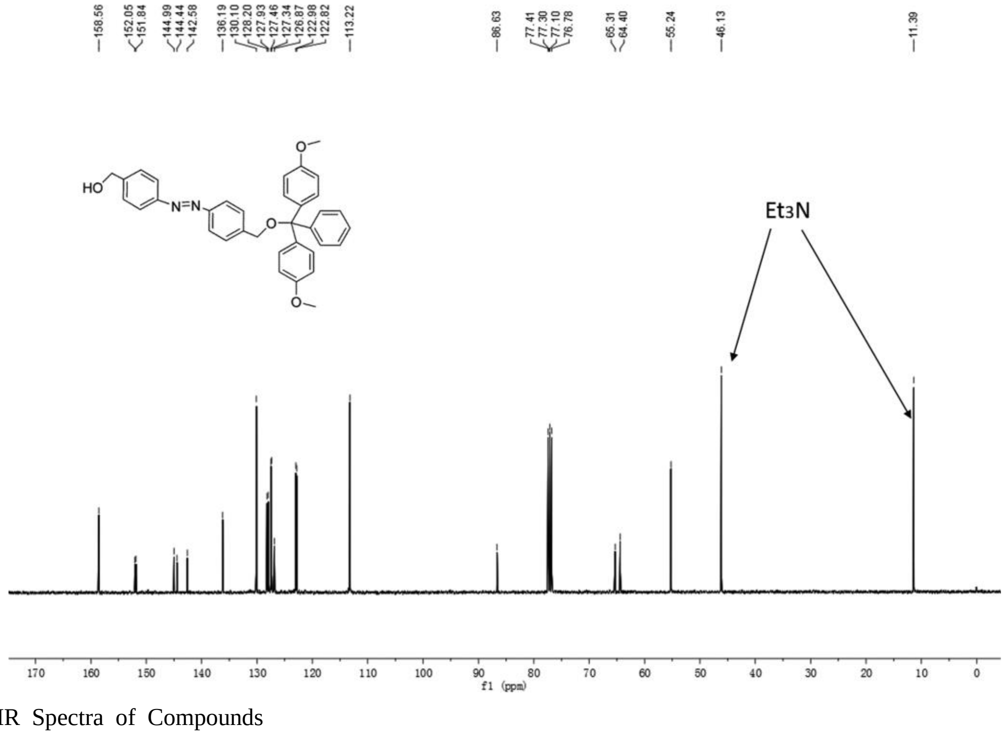

^{31}P NMR Spectra of Compounds

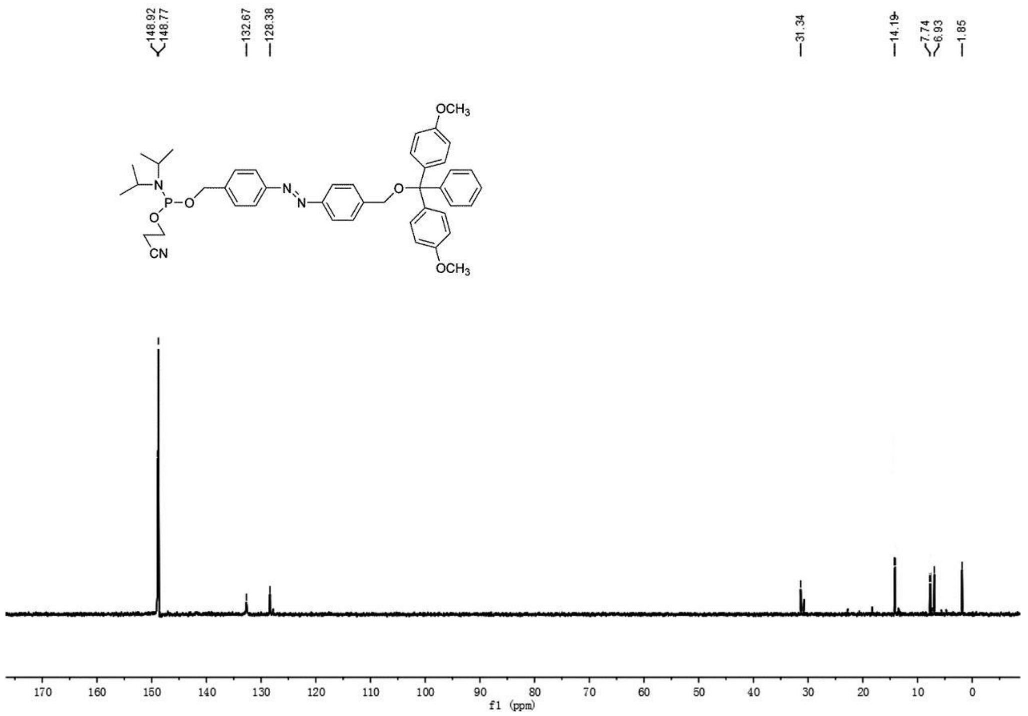

Figure S8. 1H, 13C, 31P NMR Spectra of Compounds.

Table S1. Values for ESI-MS of azobenzene linked dumbbell asODNs.

Name	Sequence	Calculated	Found
**Az1**	5'-CGTT**azo**AACGTTTCGGACCGTA**azo**TACG-3'	7952.31	7954.2
**Az2**	5'-ACGTT**azo**AACGTTTCGGACCGTA**azo**TACGG-3'	8594.73	8596.5
**Az3**	5'-AACGTT**azo**AACGTTTCGGACCGTA**azo**TACGGT-3'	9212.14	9213.0
**Az4**	5'-GTTG**azo**CAACGTTTCGGACCGTAT**azo**ATAC-3'	8569.72	8571.4
**Az5**	5'-CGTTG**azo**CAACGTTTCGGACCGTAT**azo**ATACG-3'	9188.11	9190.1
**Az6**	5'-ACGTTG**azo**CAACGTTTCGGACCGTAT**azo**ATACGG-3'	9830.53	9832.1
**Az7**	5'-TTGG**azo**CCAACGTTTCGGACCGTATT**azo**AATA-3'	9187.13	9188.2
**Az8**	5'-GTTGG**azo**CCAACGTTTCGGACCGTATT**azo**AATAC-3'	9805.52	9807.1
**Az9**	5'-CGTTGG**azo**CCAACGTTTCGGACCGTATT**azo**AATACG-3'	10423.91	10426.1

Figure S9 ESI-MS spectra of azobenzene linked dumbbell asODNs.

Az1

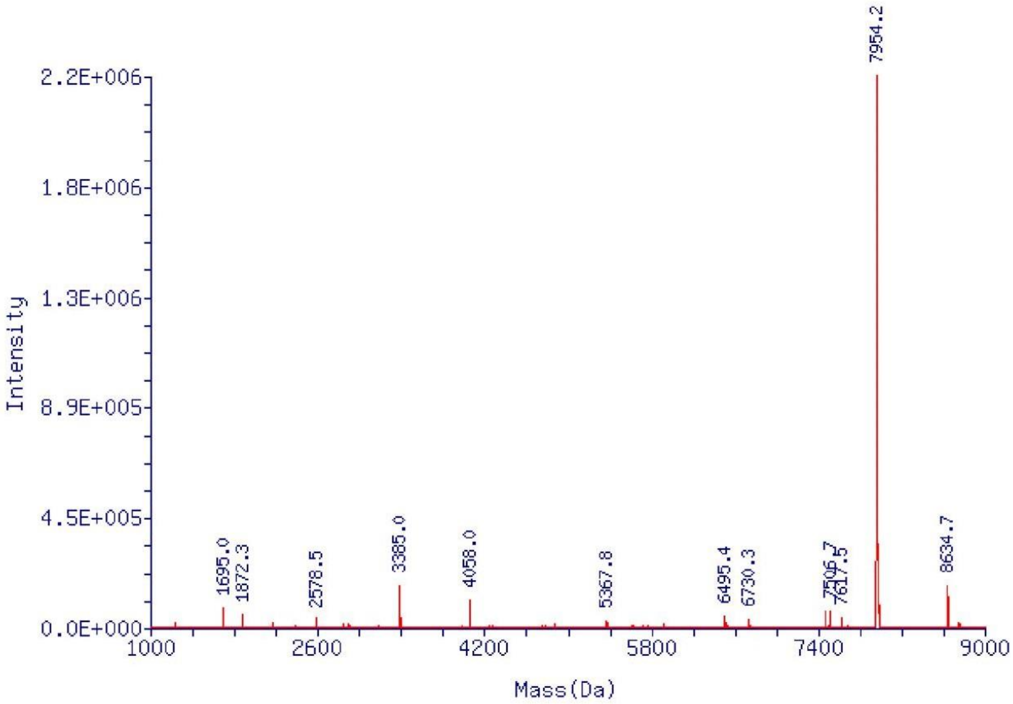
7954.2
2.2E+006
1.8E+006
1.3E+006
8.9E+005
4.5E+005
0.0E+000
Intensity
1695.0
1872.3
2578.5
3385.0
4058.0
5367.8
6495.4
6730.3
8634.7
1000
2600
4200
5800
7400
9000
Mass(Da)

Az2

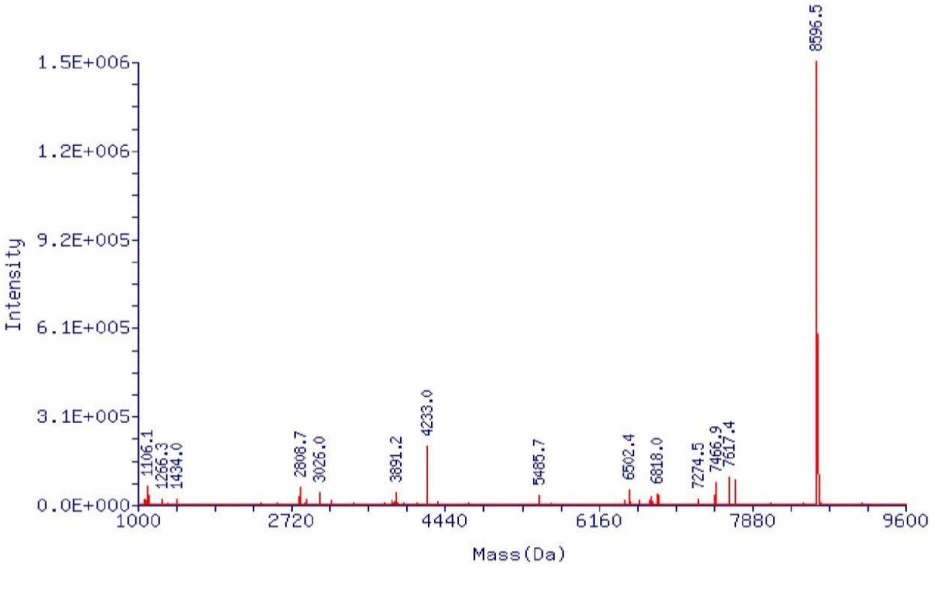
8596.5
1.5E+006
1.2E+006
9.2E+005
6.1E+005
3.1E+005
0.0E+000
Intensity
1106.1
1266.3
1434.0
2808.7
3026.0
3891.2
4233.0
5485.7
6502.4
6818.0
7274.5
7466.9
7617.4
1000
2720
4440
6160
7880
9600
Mass(Da)

Az3

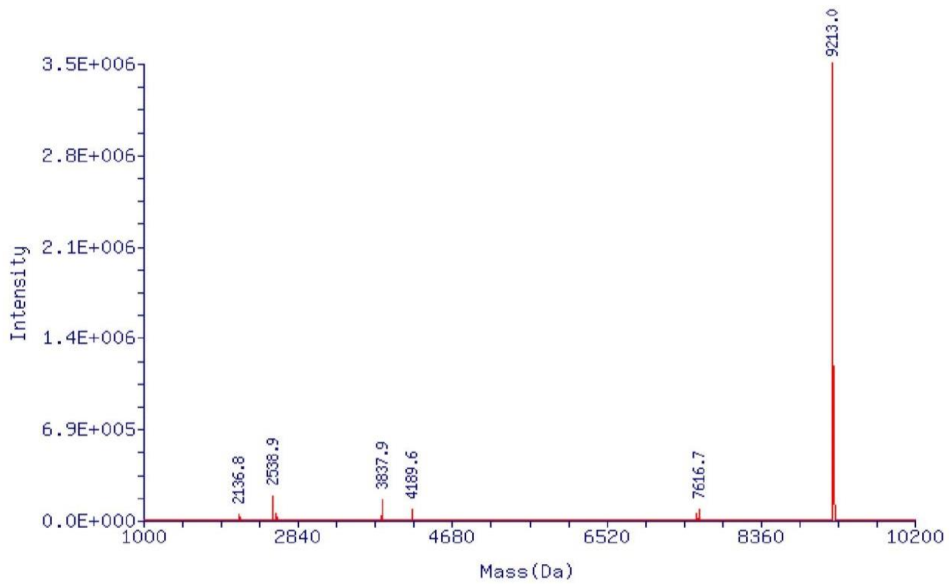
9213.0
3.5E+006
2.8E+006
2.1E+006
1.4E+006
6.9E+005
0.0E+000
Intensity
2136.8
2538.9
3837.9
4189.6
7616.7
1000
2840
4680
6520
8360
10200
Mass(Da)

Az4

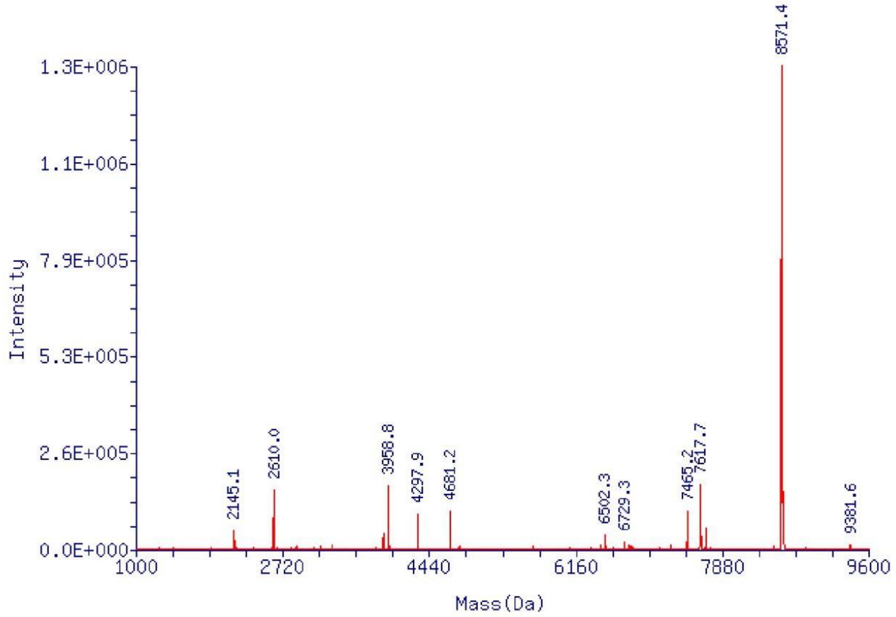
8571.4
1.3E+006
1.1E+006
7.9E+005
5.3E+005
2.6E+005
0.0E+000
Intensity
2145.1
2610.0
3958.8
4297.9
4681.2
6502.3
6729.3
7465.2
7617.7
9381.6
1000
2720
4440
6160
7880
9600
Mass(Da)

Az5

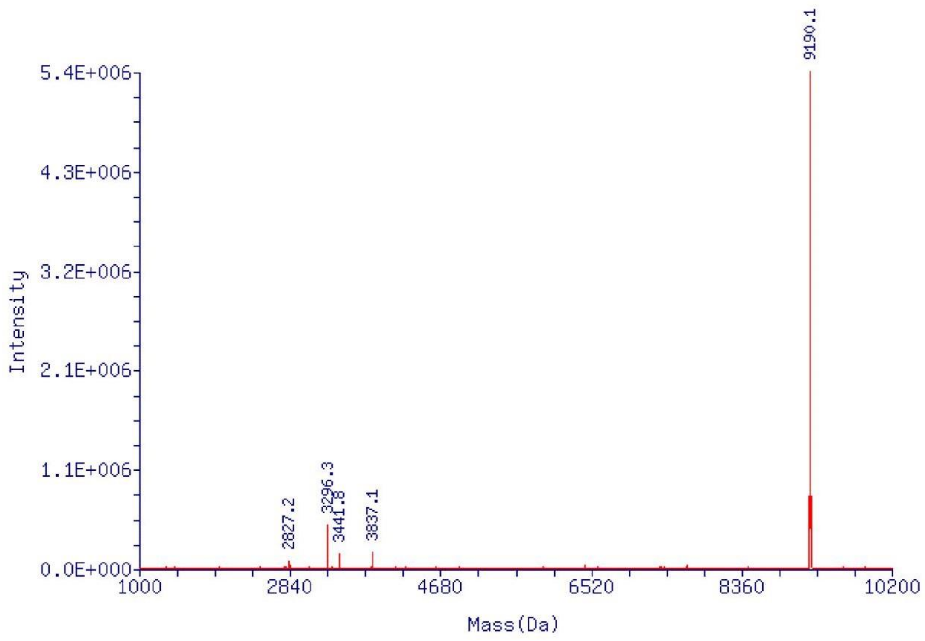
9190.1
5.4E+006
4.3E+006
3.2E+006
2.1E+006
1.1E+006
0.0E+000
Intensity
2827.2
3296.3
3441.8
3837.1
1000
2840
4680
6520
8360
10200
Mass(Da)

Az6

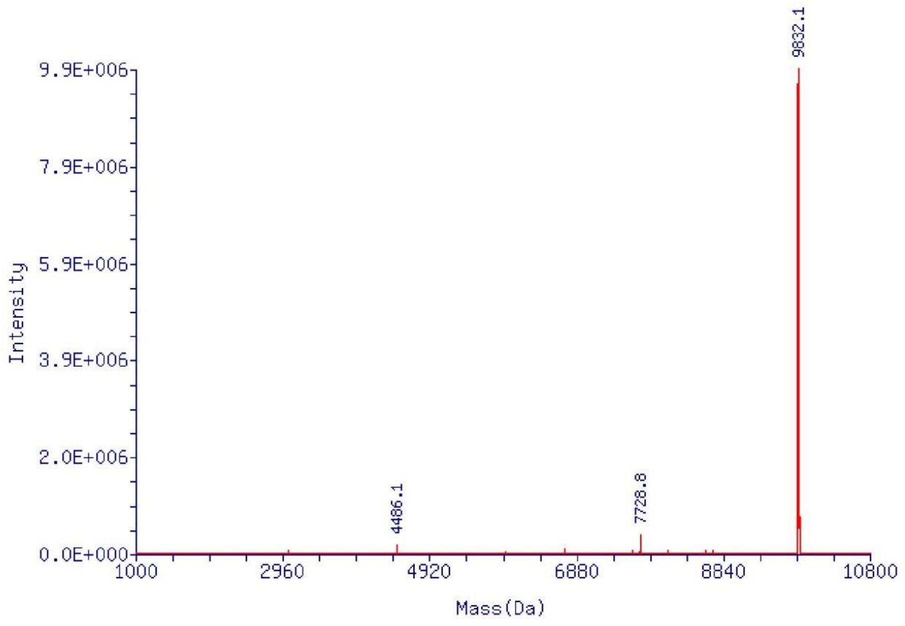
9832.1
9.9E+006
7.9E+006
5.9E+006
3.9E+006
2.0E+006
0.0E+000
Intensity
4486.1
7728.8
1000
2960
4920
6880
8840
10800
Mass(Da)

Az7

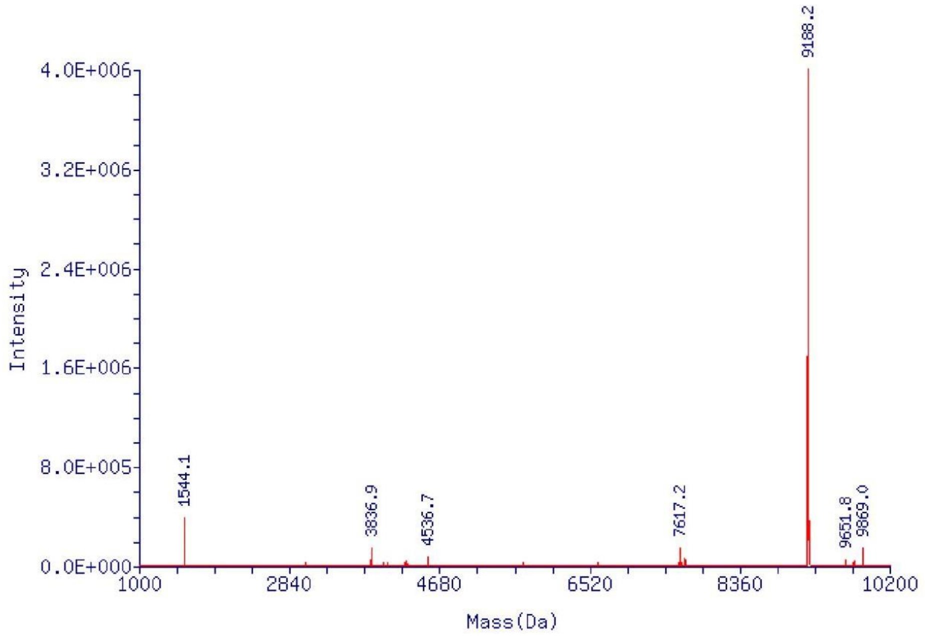

9188.2
4.0E+006
3.2E+006
2.4E+006
1.6E+006
8.0E+005
0.0E+000
Intensity
1544.1
3836.9
4536.7
7617.2
9651.8
9869.0
1000
2840
4680
6520
8360
10200
Mass(Da)

Az8

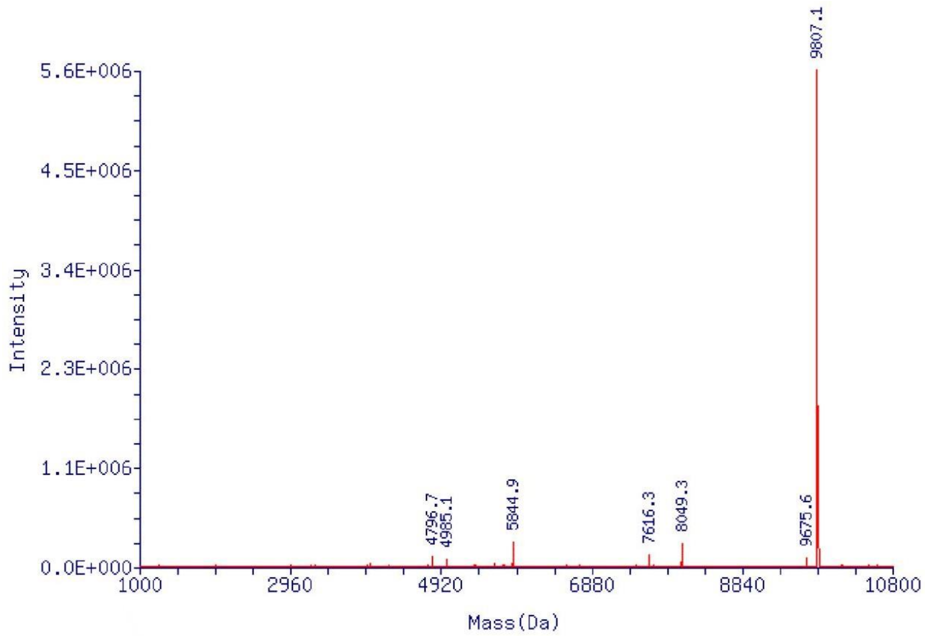

9807.1
5.6E+006
4.5E+006
3.4E+006
2.3E+006
1.1E+006
0.0E+000
Intensity
4796.7
4985.1
5844.9
7616.3
8049.3
9675.6
1000
2960
4920
6880
8840
10800
Mass(Da)

Az9

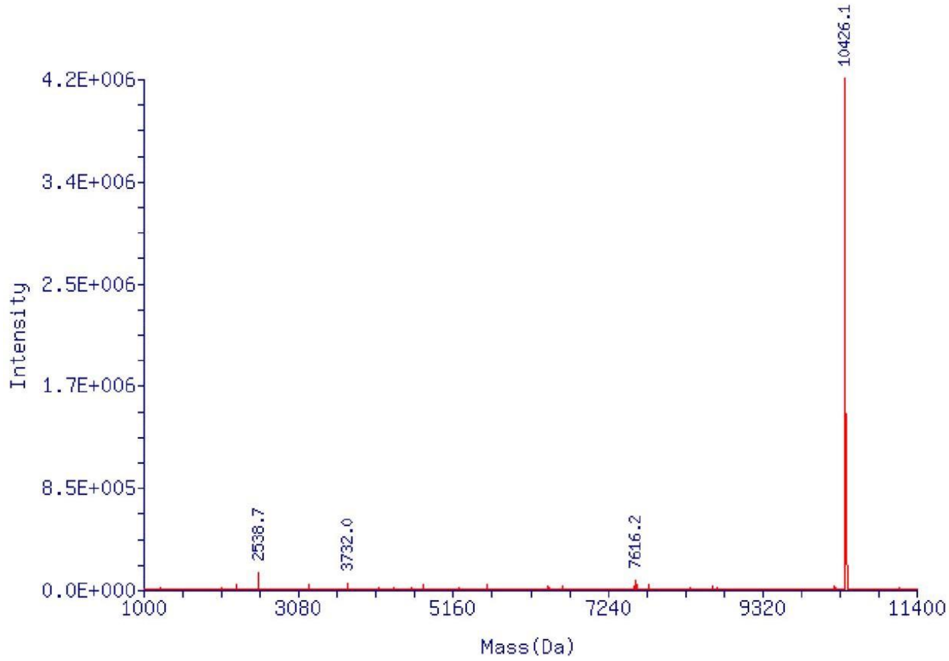

10426.1
4.2E+006
3.4E+006
2.5E+006
1.7E+006
8.5E+005
0.0E+000
Intensity
2538.7
3732.0
7616.2
1000
3080
5160
7240
9320
11400
Mass(Da)
